# Clinical characteristics of children with airway malacia complicated by pneumonia

**DOI:** 10.1186/s12879-021-06603-9

**Published:** 2021-09-03

**Authors:** Ting Wang, Qiuyan Xu, Ge Dai, Yu Hong, Zhengrong Chen, Min Lu, Yongdong Yan, Wujun Jiang

**Affiliations:** 1 Department of Respiratory Medicine, Children’s Hospital of Soochow University, Suzhou, China; 2 Department of Pediatrics, The Affiliated Suzhou Science & Technology Town Hospital of Nanjing Medical University, Suzhou, China; 3 Branch of Science & Technology Town, Children’s Hospital of Soochow University, Suzhou, China

**Keywords:** Airway malacia, Pneumonia, Chidren, Clinical characteristics

## Abstract

**Background:**

Airway malacia is an important cause of noisy breathing, recurrent wheezing and respiratory infections, chronic coughing, and episodes of respiratory distress in young children. As the clinical manifestations of airway malacia are not common, many clinicians have insufficient understanding of this disease. So the purpose of this study is to summarize the pathogenic bacteria and clinical manifestations of airway softening complicated with pneumonia in children.

**Methods:**

Children hospitalized with airway malacia complicated by pneumonia were eligible for enrollment from January 1, 2013 to December 31, 2019. Medical records of patients were reviewed for etiology, clinical characteristics, and laboratory examination results.

**Results:**

A total of 164 pneumonia patients with airway malacia were admitted. The male-to-female ratio was 3:1. The age of patients ranged from 1 month to 4 years old. The median age was 6 (3–10) months. The most commonly detected pathogen were *Mycoplasma pneumoniae* (25/164, 15.24%), *Streptococcus pneumoniae* (18/164, 10.98%), and respiratory syncytial virus (16/164, 9.76%). Common signs among the 164 patients with confirmed airway malacia included cough (98.78%), wheezing (67.07%), fever (35.37%), intercostal retractions (23.17%), dyspnea (10.98%), cyanosis (11.11%), and crackles (50%). Compared with those without airway malacia, the incidence of premature delivery and mechanical ventilation was higher, and the duration of symptoms before admission (median, 13.5 d) and hospital stay (median 10.0 d) were longer. Of the children with pneumonia, 11.59% of those with airway malacia required supplemental oxygen compared with 4.88% of those without airway malacia (p < 0.05).

**Conclusion:**

The median age of children with airway malacia was 6 months. The most common pathogen in patients with airway malacia complicated by pneumonia was *Mycoplasma pneumoniae*. Patients with airway malacia complicated by pneumonia often presented with a longer disease course, more severe symptoms, and had delayed recovery.

## Background

Airway malacia is a disease referring to an excessive collapse of the airway, which is caused by disproportionate laxity of the posterior wall (pars membranacea) or compromised cartilage integrity [1]. This concept was first proposed by Baxter and Dunbar in 1963. Airway malacia could occur in isolation, or in association with other congenital or acquired conditions [2]. It is an important cause of noisy breathing, recurrent wheezing and respiratory infections, chronic coughing, and episodes of respiratory distress in young children [3, 4]. In a study by Masters et al. [5], children with airway malacia had higher respiratory illness frequency, disease severity, likelihood of a significant cough, and tendency for a delayed recovery.

In a retrospective study, the incidence of airway malacia was at least 1/2100 [6]; however, at present, the exact incidence and prevalence of airway malacia remains uncertain. As the clinical manifestations of airway malacia—which cannot be diagnosed using routine examinations such as blood examination, imaging, or lung function tests—are not common and fiber optic bronchoscopy is limited in its application, many clinicians have insufficient understanding of this disease. This study aimed to summarize the pathogens and clinical presentation of pneumonia in children with airway malacia, and to compare these with those in children without airway malacia to provide a scientific basis for making early diagnoses and identifying reasonable treatments.

## Methods

### Patients

We performed a prospective observational cohort study of consecutive patients. This prospective study was conducted in patients aged < 4 years old with chronic respiratory symptoms or recurrent pneumonia who underwent fiber optic bronchoscopy and were admitted to the Department of Respiratory Medicine in the Children’s Hospital of Soochow University between January 1, 2013 and December 2019. The clinical definition of pneumonia is that children often present with fever, tachypnea, and other signs of respiratory distress, and can have symptoms including tachypnea, cough, dyspnea, retractions, grunting, hypoxemia, abdominal pain, or lethargy, and lung auscultation may appear decreased breath sounds, crackles, rales, or wheezing [7]. Chronic respiratory symptoms were defined as cough, stridor, or wheeze present for ≥ 3 weeks [5]; recurrent pneumonia was defined as at least two pneumonia episodes within the past 1 year [8]. The exclusion criteria for our study were: (1) patients with incomplete clinical data; (2) patients with heredity metabolic diseases, neurological disorders, congenital heart disease, and/or immunodeficiency; and (3) patients with evidence suggesting that wheezing was caused by non-infectious factors, such as bronchial foreign bodies and tuberculosis.

Patients with chronic respiratory symptoms or recurrent pneumonia who underwent fiberoptic bronchoscopy were classified into either the airway malacia group or no airway malacia group, and the ratio of the sizes of the two groups was 1:1. Airway malacia is weakness of the trachea and/or bronchial deformity at the end of expiration leading to a reduction in the diameter of the airway of more than 1/3. The airway malacia group consisted of patients with airway malacia seen on bronchoscopy. The no airway malacia group consisted of patients with no airway malacia seen on bronchoscopy. The study was approved by the Ethics Committee of Children’s Hospital of Soochow University.

### Bronchoscopy and bronchoalveolar lavage fluid (BALF) collection

The fiberoptic bronchoscopy and BALF collection procedure has been described previously [9]. The collected BALF was used for respiratory pathogen analysis (10 types of viruses and bacteria including *Mycoplasma pneumoniae* [MP]).

According to the part of airway with malacia on bronchoscopy, airway malacia was classified into tracheomalacia, bronchomalacia, or tracheobronchomalacia. Tracheomalacia refers to a weakness of the trachea that could make the airway softer and more susceptible to collapse with changes in pressure. Bronchomalacia is the isolated weakness and collapsibility of one or both of the mainstem bronchi and/or their respective divisions at the lobar or segmental level without tracheal involvement. Tracheobronchomalacia is the appearance of a deformity including the whole or segments of the trachea and one or both of the mainstem bronchi.

According to the degree of malacia, the severity of malacia was divided into mild (one-third to one-half invagination), moderate (one-half to three-quarters invagination), and severe (more than three-quarters invagination) [10].

### Detection of respiratory pathogens analysis

Polymerase chain reaction(PCR) was used to detect respiratory syncytial virus infection (RSV), human metapneumovirus (HMPV), human bocavirus (HBoV), and human rhinovirus (HRV),influenza virus A (IVA), influenza virus B (IVB), parainfluenza virus (PIV) I, PIV II, PIV III, adenovirus (ADV), and MP in BALF [11].

Bacteria were identified using quantitative analysis by inoculating BALF onto elected media for 18–24 h. Bacterial growth > 10^3^ colony-forming units/mL was considered significant [12].

### Data collection

Data regarding personal history, demographics, clinical characteristics, and laboratory examination results were documented after admission. Demographic and clinical characteristics included age, sex, length of hospital stay, and requirement for supplemental oxygen. Laboratory examination results including white blood cell (WBC) count, neutrophil count, C-reactive protein (CRP) level, and detection of common viruses were recorded.

### Statistical analyses

Statistical analyses were conducted using SPSS 26.0 (IBM, SPSS, Chicago, IL, USA).

Descriptive continuous outcome variables were shown as medians (25% to 75%).Comparison of quantitative variables among different groups were performed using one-way analysis of variance or the Kruskal–Wallis test when the variance was the same in different groups. Comparison of the frequency distribution was performed using the chi-square test; p < 0.05 was considered as significant. A sample size estimation was calculated using Power Analysis and Sample Size (PASS) software. Based on a likely sample proportion of interest variable having the tested trait (P) of 15% [10], with 90% confidence (α = 0.1) and a 10% margin of error of the estimate, the minimum required sample size was n = 157.

## Results

### Demographic characteristics

A total of 164 patients with airway malacia complicated by pneumonia were recruited to the study (Table 1). Of these 164 patients, 123 (75%) were male and 41 (25%) were female. The male-to-female ratio was 3:1. The age of the patients ranged from 1 month to 4 years old. Ninety (54.88%) patients were aged ≤ 6 months, 42 (25.61%) patients were aged 6–12 months, and 32 (19.51%) patients were aged ≥ 12 months, The median age was 6 (3–10) months.

**Table 1:** Airway malacia in patients with pneumonia

Patient characteristics	Case
Sex (male/female)	123/41
Mean age (months)(IQR)	6 (3 ~ 10)
Age group n (%)	
≤ 6 months	90 (54.88%)
6 ~ 12 months	42 (25.61%)
≥ 12 months	32 (19.51%)
Part of malacia	
Tracheomalacia	34 (20.73%)
Bronchiomalacia	88 (53.66%)
Tacheobronchiomalacia	42 (25.61%)
Degree of malacia	
Mild malacia	67 (40.85%)
Moderate malacia	71 (43.29%)
Severe malacia	26 (15.86%)
Range of malacia	
Single malacia	95 (57.93%)
Double malacia	37 (53.62%)
Multiple malacia (≥ 3)	32 (46.38%)

Descriptive continuous outcome variables were shown as medians (25 to 75%)

There were 34 (20.73%), 88 (53.66%), and 42 (25.61%) patients with tracheomalacia, bronchomalacia, and tracheobronchomalacia, respectively. Of the 88 children with bronchomalacia, 44 (50%) had right-sided disease, 25 (28.41%) had left-sided disease, and 19 (21.59%) had bilateral disease. In all cases of pneumonia with airway malacia, 24/164 (14.63%) were complicated by other airway dysplasia, most frequently laryngomalacia (12/24, 50%), followed by bronchial stenosis (6/24, 25%), abnormal opening of the bronchus (4/24, 16.67%), and tracheal bronchus (2/24, 8.33%); 40.85% (67/164) of patients had mild airway malacia, 43.29% (71/164) of patients had moderate airway malacia, and 15.86% (26/164) of patients had severe airway malacia.

### Pathogens

**Fig. 1:**
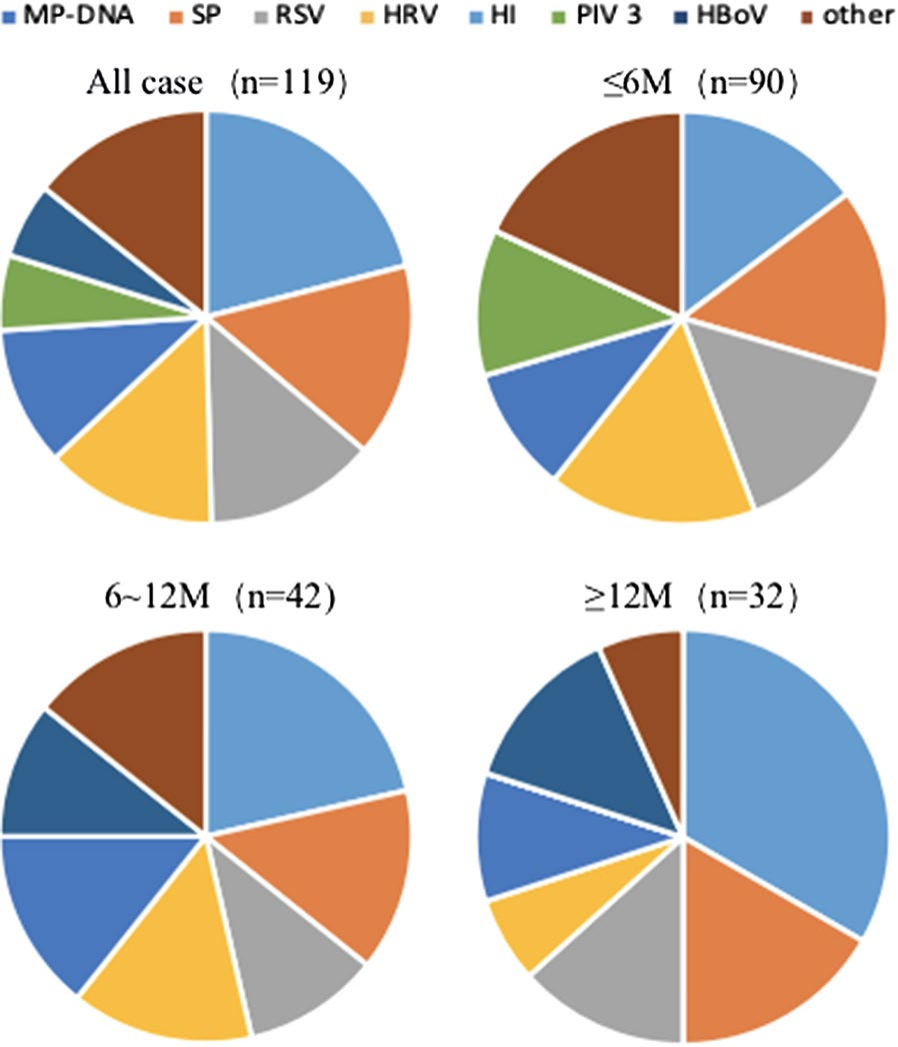
Distribution of pathogens identified from patients with airway malacia in BALF, stratified by age

Overall, because more than one pathogen could be detected in a patient, a total of 119 different pathogens were detected (Fig. 1). The most commonly detected pathogen was MP (25/164, 15.24%). Meanwhile, among the detected viruses, RSV (17/164, 10.37%) was the most common, followed by HRV (15,9.15%), PIV3 (7/164, 4.27%), HBoV (7/164, 4.27%), ADV (2/164, 1.22%), PIV 1 (1/164, 0.61%), and hMPV (1/164, 0.61%).

**Fig. 2:**
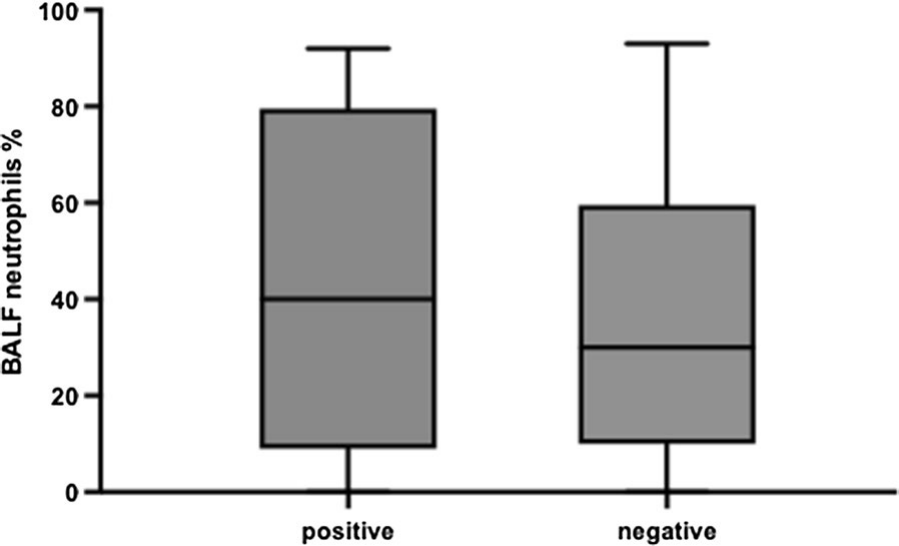
Percentage of neutrophils in BALF of patients that BALF culture was positive and negative. P > 0.05

**Fig. 3:**
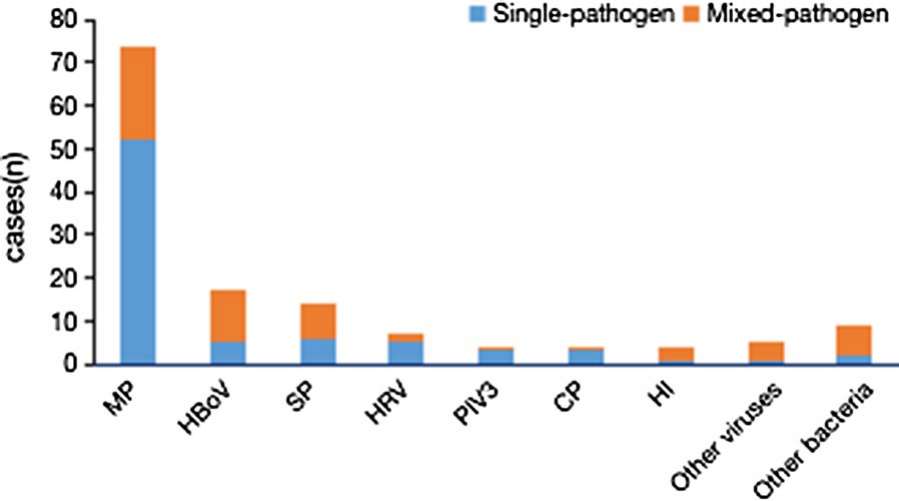
Pathogens identified from patients with airway malacia

**Fig. 4:**
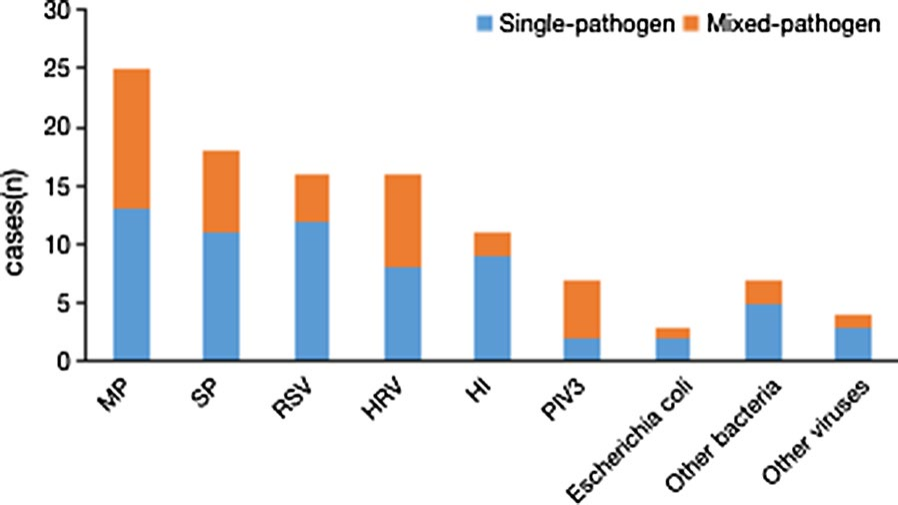
Pathogens identified from patients with non-airway malacia

A positive BALF culture was obtained in all children with airway malacia; 26.83% (44/164) BALF samples had a positive bacterial culture: 10.98% (18/164) *Streptococcus pneumoniae* (SP), 8.54% (14/164) *Haemophilus influenzae* (HI), 1.83% (3/164) *Escherichia coli*, 1.22% (2/164) *Staphylococcus aureus*, 1.22% (2/164) *Enterobacter aerogenes*, 1.22% (2/164) *Klebsiella pneumoniae*, 0.61% (1/164) coagulase-negative staphylococci, 0.61% (1/164) *Acinetobacter baumannii*, 0.61% (1/164) *Moraxella catarrhalis*, and 0.61% (1/164) *Acinetobacter junii*. Patients with a positive BALF culture had a higher median neutrophil percentage in the BALF (Fig. 2); however, this difference was not statistically significant. The distribution of bacterial and viral etiology in children with and without malacia was in Figs. 3 and 4.

In patients (n = 90) aged ≤ 6 months, 61 pathogens were detected; the most commonly detected viral pathogens were HRV (10/61, 16.39%), RSV (9/61, 14.75%), and PIV3 (7/61, 11.48%). In patients aged 6–12 months (n = 42) and ≥ 12 months (n = 32), the most commonly detected viral pathogens were RSV (3/28, 10.71% and 4/30, 13.33%, respectively), HRV (4/28, 14.29% and 2/30, 6.67%, respectively), and HBoV (3/28, 10.71% and 4/30, 13.33%, respectively). The main bacterial pathogens in children of all ages were SP and HI.

### Clinical characteristics of pneumonia children with airway malacia and Non-airway malacia

In the airway malacia group, the incidence of premature delivery and mechanical ventilation was higher than that in the non-airway malacia group. Children with pneumonia with airway malacia had a longer duration of symptoms before admission (median, 13.5 d) and longer hospital stay (median, 10.0 d) than children with pneumonia without airway malacia. A greater proportion of children with pneumonia with airway malacia required supplemental oxygen than children with pneumonia without airway malacia (11.59% vs. 4.88%. respectively, p < 0.05). Children with pneumonia with airway malacia were significantly more likely to have wheezing (67.07%), laryngeal stridor (32.93%), dyspnea (10.98%), cyanosis (11.11%), and intercostal retraction (23.17%); all of these were statistically different between the two groups (all p < 0.05). Children with airway malacia had a higher neutrophil count than children without airway malacia, while there was no significant difference between the two groups with respect to the WBC count or CRP level (p > 0.05) (Table 2).

**Table 2:** Clinical features of pneumonia children hospitalized with or without airway malacia

Clinical features	Airway malacia (n = 164)	Non-airway malacia (n = 164)	*P*
General features			
Gender (male/female)	123/41	114/50	0.267
Mean age (year-old)	6 (3 ~ 10)	6 (3 ~ 9)	0.498
Personal history			
Premature delivery n (%)	27 (16.46)	11 (6.71)	0.006
Mechanical ventilation n (%)	10 (6.10)	2 (1.22)	0.019
Length of stay (day)(IQR)	10 (7.25 ~ 15)	7 (6 ~ 8)	< 0.001
Symptom duration prior to admission (day)(IQR)	13.5 (4 ~ 30)	6.5 (4 ~ 10)	< 0.001
Requirement for supplemental oxygen (%)	19 (11.59)	8 (4.88)	0.027
Clinic presentation			
Cough	162 (98.78)	163 (99.39)	1.000
Wheezing	110 (67.07)	79 (48.17)	< 0.001
Fever	58 (35.37)	63 (38.41)	0.567
Laryngeal stridor	54 (32.93)	17 (10.37)	< 0.001
Dyspnea	18 (10.98)	8 (4.88)	0.041
Cyanosis	11 (11.11)	2 (1.22)	0.011
Retractions	38 (23.17)	6 (3.66)	< 0.001
Crackles n (%)	82 (50.00)	90 (54.88)	0.376
Laboratory findings			
WBC count(× 10^9^/L) (IQR)	10.55 (7.61 ~ 14.99)	10.34 (7.56 ~ 12.89)	0.212
N count (× 10^9^/L) (IQR)	3.67 (2.32 ~ 5.58)	3.06 (1.86 ~ 5.01)	0.013
CRP count > 8 mg/L [n (%)]	24 (14.63)	31 (18.90)	0.301

Analysis of variance was done for both groups, and variances are equal. Analysis of variance was done for both groups, and variances are equal

### Clinical and laboratory characteristics of different age groups of children hospitalized with airway malacia complicated by pneumonia

To evaluate the difference among different age groups of children with airway malacia complicated by pneumonia, we divided the children into three age groups (Table 3). There was no significant difference in sex between the different age groups. The common characteristics among the 164 confirmed patients were cough (98.78%), wheezing (67.07%), fever (35.37%), retractions (23.17%), dyspnea (10.98%), cyanosis (11.11%), and crackles (50%); fever was more commonly detected in older children and wheezing was more commonly detected in children aged 6–12 months, while younger patients were more likely to have intercostal retractions. Other characteristics showed no significant difference among the three age groups (p > 0.05). Older children were more likely to have a higher neutrophil count than younger children (p < 0.05). The incidence of MP was higher among children aged > 6 months than among younger children and was highest among children aged ≥ 12 months. The incidence of different viruses and bacteria were similar across age groups.

**Table 3:** Clinical features of pneumonia children hospitalized with airway malacia in different age groups

Clinical features	≤ 6 months (n = 90) n (%)	6 ~ 12 months (n = 42) n (%)	≥ 12 months (n = 32) n (%)	*P*
Female sex	26 (28.89)	7 (16.67)	8 (25)	0.32
Clinic presentation				
Cough	89 (98.89)	41 (97.62)	32 (100)	0.703
Wheezing	51 (56.67)	36 (85.71)	23 (71.88)	0.003
Fever	19 (21.11)	19 (45.24)	20 (62.50)	< 0.01
Dyspnea	14 (15.56)	3 (7.14)	1 (3.13)	0.075
Cyanosis	8 (8.89)	1 (2.38)	2 (6.25)	0.320
Retractions	27 (30.00)	9 (21.43)	2 (6.25)	0.023
Crackles	47 (52.22)	19 (45.24)	17 (53.13)	0.719
Laboratory findings				
WBC count (× 10^9^/L(IQR))	9.98 (7.23 ~ 15.92)	10.86 (7.89 ~ 13.49)	12.06 (8.36 ~ 15.26)	0.538
Neutrophils count (× 10^9^/L)(IQR)	3.2 (2.25 ~ 4.83)	4.14 (2.15 ~ 6.46)	4.75 (2.99 ~ 7.94)	0.024
CRP count > 8 mg/L [n (%)]	10 (11.11)	8 (19.05)	6 (18.75)	0.371
Pathogens				
Virus	29 (32.22)	10 (23.81)	11 (34.38)	0.538
Bacteria	23 (25.56)	11 (26.19)	10 (31.25)	0.818
MP	9 (10.00)	6 (14.29)	10 (31.25)	0.026

Analysis of variance was done for both groups, and variances are equal. Analysis of variance was done for both groups, and variances are equal

## Discussion

Airway malacia refers to an excessive increase in compliance of the trachea, such that the airway is more susceptible to dynamic and/or static collapse. Jacobos IJ et al. reported that approximately 11–15% of children who undergo bronchoscopy have airway malacia [13], while the rate of occurrence in China is 16.7–22.1% [10, 14]. This study found that about 20% of children with long-term cough, wheezing, and recurrent infections who underwent bronchoscopy had airway malacia between January 2013 and November 2019, which is consistent with domestic research but higher than that reported in foreign research. The difference might be due to differences in diagnostic standards. In most foreign countries, the diagnostic criterion is a reduction in the airway diameter of more than 1/2 [3], while in China the diagnostic criterion is a more than 1/3 reduction in airway diameter, which results in a higher diagnosis rate in China than in foreign countries. In addition, the wide application of bronchoscopy in China in recent years has led to increasing numbers of children being diagnosed early.

In the present study, 75% of children with airway malacia were male, which was consistent with reports that of children with airway malacia, 58–82% are male [10]; 80.49% of children with airway malacia were < 1 year old, and the median age was 6 months, which is consistent with Deacon et al. who reported that the median age of children with airway malacia was 6.8 months [15].

Airway malacia could be divided into primary and secondary malacia in origin.

Primary airway malacia is related to premature delivery, malnutrition during pregnancy, and calcium deficiency. Hysinger et al. [16] reported that moderate or severe airway abnormalities were identified in 32/117 (27.3%) preterm infants.

In the present study, we found the incidence of premature delivery in children with airway malacia was 16.46%, which was higher than 6.71% of children without airway malacia. In premature infants, airway compliance is higher, the ratio of cartilage to airway smooth muscle is lower, tracheal muscular tension is low, and the smaller airway is easier to narrow.

The clinical symptoms associated with airway malacia are often atypical, mainly including recurrent wheezing, persistent or recurrent pneumonia, chronic cough, and dyspnea, among others. Holingert et al. [17] suggested that stridor and barking cough were the most common symptoms. Tan et al. [4] indicated that reflex apnea is the most severe clinical symptoms. In mild airway malacia, a collapsed tracheobronchial wall during exhalation could prevent the effective clearance of secretions and cause occasional respiratory infections. In moderate airway malacia, wheezing is more frequently associated with lung respiratory tract infections. In severe malacia, the affected children could develop upper airway obstruction accompanied by cyanosis, expiratory stridor, and apnea. In the present study, symptom duration before admission and length of hospital stay were greater in children with airway malacia than those without airway malacia. This suggest that patients with pneumonia with airway malacia often present with slower disease progression. In addition, we also found that wheezing, laryngeal stridor, dyspnea, anhelation, cyanosis, and intercostal retractions were more common in patients with airway malacia. Furthermore, airway malacia was associated with increased severity relative to the disease in patients without airway malacia, which was consistent with a previous study where patients with airway malacia had a tendency for delayed recovery and more serious disease [5].

Previous reports on the etiology of pneumonia combined with airway malacia are very rare. Recognizing the pathogenic distribution of airway malacia complicated by pneumonia is of great significance to clinical treatment. In the present study, we found that the most common pathogen was MP, which is consistent with previous studies [12, 18, 19]. At the same time, we found that there was no significant difference in the detection rate of pathogens, including viruses, MP, and bacteria, in children with and without airway malacia. This result differed from that of our previous report [12]. This may be due to differences in the inclusion criteria. In this study, patient enrollment was limited to those < 4 years old; in contrast, the study of Wang Yu-qing et al. did not limit the age of enrolled patients. Considering that the typical age of patients with airway malacia is < 4 years old, we only enrolled patients < 4 years old. Some studies [10, 20] have suggested that airway malacia could be improved or even eliminated when airway compression is removed or if inflammation is controlled promptly. Therefore, timely bronchoscopy and early identification of pathogens and timely treatment of infection are very important.

Our study has the following advantages. Firstly, there are few studies on airway malacia, especially in southern China. Secondly, we made a detailed record and discussion on the clinical characteristics and etiological analysis of patients with malacia. Especially on the etiology, there are only a few studies on this aspect at home and abroad. At the same time, our study also has potential limitations. Firstly, some patients may have been treated with antibiotics before admission and the interval between fiberoptic bronchoscopy and admission varied among patients, which may have led to BALF specimens that were collected after different times with respect to antibiotic treatment and may have a certain effect on the results. Secondly, use of direct immuno staining is less sensitive than PCR, culture for bacteria may miss important pathogens.

## Conclusion

In conclusion, the median age of children with airway malacia was 6 months. Patients with airway malacia were more commonly male; bronchomalacia was the main type of disease, with malacia of the right bronchus being the most frequently reported subtype. Single airway malacia was more common than bilateral malacia. The most common pathogen was MP, and there was no significant difference in the detection rate of pathogens in children with and without airway malacia. Patients with pneumonia with airway malacia often present with slower disease progression, have a tendency for delayed recovery, and have more serious disease. Therefore, it is necessary to perform fiberoptic bronchoscopy in younger patients with pneumonia with a longer disease course, severe symptoms, and delayed recovery for confirmation of the diagnosis and to allow timely treatment.

## Data Availability

The datasets used and/or analysed during the current study are available from the corresponding author on reasonable request.

## Abbreviations

BALF: Bronchoalveolar lavage fluid
MP: *Mycoplasma pneumonia*
PCR: Polymerase chain reaction
RSV: Respiratory syncytial virus infection
HMPV: Human metapneumovirus
HBoV: Human bocavirus
HRV: Human rhinovirus
IVA: Influenza virus A
IVB: Influenza virus B
PIV: Parainfluenza virus
ADV: Adenovirus
WBC: White blood cell
CRP: C-reactive protein
